# The presentation pattern and surgical strategies in bronchopulmonary carcinoid tumors: a multicenter experience in a low-income country

**DOI:** 10.3389/fsurg.2024.1399999

**Published:** 2024-08-22

**Authors:** Workneh Tesfaye Deme, Seyoum Kassa Merine, Desalegn Fekadu Wadaja, Abdela Hayato Gemeda, Meklit Tamrat Demissie, Mahlet Tesfaye Bahta, Wondu Reta Demissie

**Affiliations:** ^1^ Cardiothoracic Unit, Department of Surgery, College of Health Science, Addis Ababa University, Addis Ababa, Ethiopia; ^2^ Department of Surgery, Faculty of Medical Sciences, Institute of Health, Jimma University, Jimma, Ethiopia; ^3^ Department of Surgery, Adama Hospital Medical College, Adama, Ethiopia; ^4^ Department of Biomedical Sciences, Faculty of Medical Sciences, Institute of Health, Jimma University, Jimma, Ethiopia

**Keywords:** bronchopulmonary carcinoid tumor, presentation pattern, surgical strategies, low-income country, multicenter study

## Abstract

**Background:**

Bronchopulmonary carcinoid tumors include typical and atypical carcinoids, with typical carcinoids accounting for 80%–90% of these types of tumor. The primary curative treatment for these tumors is surgical resection. To our knowledge, there are limited studies on the presentation patterns and treatment strategies of bronchopulmonary carcinoid tumors in Africa.

**Objective:**

To determine the presentation patterns and surgical strategies in bronchopulmonary carcinoid tumors in patients treated at multicenters in Ethiopia from January 2018 to December 2023.

**Materials and methods:**

A 5-year retrospective cross-sectional study was conducted using medical records and pathology record reviews of patients operated on in Tikur Anbessa Specialized Hospital, Menelik II Hospital, and Saint Peter's Specialized Hospital from 1 January 2018 to 31 December 2023. The completeness of the data was checked before being entered into EpiData version 4.6.1, and analysis was conducted using SPSS version 29. Logistic regression was applied to depict the association of the histological pattern with its predictors. A *P*-value of <0.05 was considered significant for the association of variables.

**Results:**

A total of 62 patients with bronchopulmonary carcinoid tumors were included in the study with a mean age of 35.29 ± 12.26 years ranging from 14 to 67 years, in which more than half [37 (56.5%)] were females, with a male-to-female ratio of 1:1.3. The majority of the patients were non-smokers (90.3%) and symptomatic (98.4%), with a mean duration of symptoms of 29.7 ± 26 months, ranging from 3 to 156 months. Nearly half of the patients (48.4%) were treated for pulmonary tuberculosis before a diagnosis of carcinoid tumor was made. The majority of the patients underwent surgery by open posterolateral thoracotomy (98.4%), and pneumonectomy was the most common (38.7%) resection performed. Typical carcinoids were observed in 85.5% of patients. Age, smoking history, duration of symptoms, location of tumors, and lymph node status were statistically associated with histological patterns.

**Recommendation:**

Based on our study findings, improving physician awareness on the clinical presentation of carcinoid tumors, training for surgeons in less invasive surgical approaches, and further nationwide studies are recommended.

## Introduction

Neuroendocrine tumors (NETs) are groups of neoplasms that arise from specialized, peptide, and amine-producing cells found in the diffuse endocrine system. They frequently occur in gastrointestinal tracts (48%), followed by the lungs (25%). Other possible sites of occurrence include the pancreas, breast, prostate, thymus, and skin (1).

Bronchopulmonary neuroendocrine tumors (BPNETs) include groups of lung neoplasms that have a distinctive basic microscopic appearance and immunohistochemistry features. The World Health Organization (WHO) Tumor Classification 2015 divides BPNETs into four subtypes: low-grade typical carcinoid tumor (TC), intermediate-grade atypical carcinoid tumor (AC), high-grade malignancies consisting of a large-cell neuroendocrine carcinoma (LCNEC), and small-cell lung carcinoma (SCLC). Even though they have different clinical behaviors, they are grouped into the same category because of their neuroendocrine features (2, 3).

Typical and atypical carcinoids are grouped as bronchopulmonary carcinoid tumors (BPCTs), which are well-differentiated BPNETs. TC is more common, accounting for 80%–90% of BPCTs, whereas AC only accounts for 20%. The distinction between TC and AC is based on the WHO tumor classification determined by a cellular mitotic rate and the presence or absence of necrosis. TC has <2 mitoses/2 mm^2^ and no necrosis, whereas AC has 2–10 mitoses/2 mm^2^ and focal necrosis. In most cases, BPCTs have very few genetic abnormalities compared with SCLC and LCNEC. Furthermore, patients with carcinoid tumors are younger and have a better prognosis. The majority of TC patients are non-smokers, whereas AC appears to be slightly more frequent among smokers (4, 5).

The incidence of these tumors has rapidly increased, which might be due to the development of tumor screening and imaging technologies. The incidence of BPCTs is higher among white people than among black people (6).

The distribution of BPCTs is mostly comparable between males and females, but in some studies, female predominance was reported and being male had a worse prognosis (7). The median age of patients diagnosed with these tumors is approximately 48 years. Anatomically, most BPCTs occur in the central bronchial tree, with TC being the dominant histological type (8, 9).

In most of the studies, patients were symptomatic at presentation. The most common presentation symptoms are non-resolving respiratory infections and hemoptysis. Typically, the centrally located tumor tends to cause infection, hemoptysis, or cough. The AC-type histological pattern tends to be associated with a larger tumor size, with an increased incidence of lymph node involvement, and distant metastasis. When laterality is considered, BPCTs are more common in the right lung (10–13).

On the other hand, other studies have found that more than half of patients treated for BPCTs are asymptomatic at the time of presentation. According to these studies, most of these tumors are found incidentally on chest x-rays, frequently appearing as isolated well-defined hilar or perihilar masses. The use of low-dose chest computed tomography (CT) scans for lung cancer screening might even increase the proportion of these patients further (14, 15).

In most patients, there is no lymph node involvement. When the distribution of lymph node positivity is considered, AC-type BPCTs are more likely to involve lymph nodes. Daddi et al. have reported lymph node metastases in approximately 30% of cases of atypical pulmonary carcinoids (16). The proportion of patients with distant metastasis also differs significantly between studies (17–20).

The clinical diagnosis of these patients usually involves a chest CT scan and bronchoscopy. The common pattern in chest CT findings varies among studies. In some studies, hilar or perihilar masses and pulmonary nodules were more frequent, whereas in other studies atelectasis and pulmonary infiltration were the dominant findings. A preoperative confirmation of carcinoid tumor with bronchoscopic biopsy was possible in most of the cases (21–23).

There is controversy about the optimum treatment of these tumors. In general, the primary and only curative treatment of BPCTs remains surgical resection. The principle of the surgical technique in these patients is a complete negative margin resection with preservation of normal lung tissue. Previously, more radical pulmonary resection was considered for atypical carcinoids but it was found that the extent of resection does not impact overall survival (24–28). Compared with bronchoplastic and lobar resection, pneumonectomy should be the least preferred option as it is associated with worse overall survival (16).

The most conventional surgical approach for BPCTs is open thoracotomy, followed by video-assisted thoracoscopic surgery (VATS) (29–31). Anatomic lobectomy was the most commonly performed surgical treatment but the proportion is different among studies. Other frequently performed resections were sleeve lobectomy, wedge resections, and pneumonectomies. Pneumonectomy was performed for a tumor located in the main bronchus with obstructive pneumonia or atelectasis, and when the tumor invaded the pulmonary vessels (32–35). In some cases, bronchoplastic procedures were also performed as a primary surgical treatment (13, 36–40).

To date, there have been limited studies conducted on the presentation patterns and surgical strategies used for the treatment of BPCTs in the African context [with scarce evidence from Egypt (36) and Tunisia (37)], where no studies have been undertaken in Ethiopia. Therefore, the purpose of this study was to explore the demographics, clinical presentations, anatomic distributions, and histological types of BPCTs in Ethiopia. The study also determined the proportion of patients with carcinoid tumors treated for another presumptive diagnosis. Ultimately, we have assessed the surgical strategies used in these patients, as well as the factors influencing histological distributions of carcinoid tumors.

## Materials and methods

### Study area and period

The study was conducted at three tertiary care hospitals in Addis Ababa, Ethiopia, from 1 January 2018 to 31 December 2023. Ethiopia, officially the Federal Democratic Republic of Ethiopia, is a landlocked country in the Horn of Africa. It shares borders with Eritrea to the north, Djibouti to the northeast, Somalia to the east and southeast, Kenya to the south, South Sudan to the west, and Sudan to the northwest. Ethiopia covers a land area of 1,112,000 km^2^ (472,000 square miles). As of 2022, its projected population is estimated to be 105.17 million, making it the second most populous country in Africa after Nigeria (38). Addis Ababa is the national capital and largest city.

The Tikur Anbessa Specialized Hospital (TASH), established in 1972, is a public teaching hospital of the Addis Ababa University, College of Health Sciences. It is the largest referral hospital in the country with 700 in-patient beds. Menelik Hospital (MH) is also a public hospital and was established in 1909; it was the first hospital in Ethiopia. Saint Paul's Specialized Hospital (SPSH) is another public hospital with a tertiary care service. All hospitals have thoracic surgery services.

### Study design

A 5-year retrospective cross-sectional study was conducted using medical records and pathologic record reviews of patients operated on during the study period.

### Population

The source population was all patients who had undergone operations in the cardiothoracic operating room at multicenter institutions in Ethiopia. The study population was all patients who had undergone surgery within the last 5 years for a diagnosis of BPCTs. Preoperative evaluation of patients included a clinical history, physical examination, chest CT scan, and/or flexible bronchoscopy. General anesthesia was given with double lumen intubation and a thoracic epidural catheter for pain management was carried out routinely. The usual surgical approach was posterolateral thoracotomy incision with muscle division. Anatomic resection was the standard surgical technique for obtaining a negative margin and sparing healthy lung parenchyma as much as possible. Pneumonectomy was performed for tumors located in the main bronchus causing obstructive pneumonia, for destroyed lung despite the tumor location, or when lobectomy was not technically possible due to excessive adhesions. Bronchoplastic procedures were reserved for tumors located centrally and completely endoluminal with normal underlying lung parenchyma.

### Inclusion and exclusion criteria

All patients who had undergone surgical treatment for a diagnosis of BPCTs in the last 5 years were included. Patients with incomplete medical records, if operation was done for recurrence, and/or those with post-resection biopsy results of noncarcinoids were excluded.

### Data collection tool/procedures

Data related to the patient's baseline characteristics and clinical profiles (presentation patterns, histopathologic patterns, and surgical treatments) were collected using a structured questionnaire prepared in English. The data were extracted from the operation room logbook, preoperative patient medical records, operation notes, pathologic records, and post-operative follow-up records. Three resident physicians were involved in data collection after adequate training was provided.

### Ethical consideration

Data collection was commenced after ethical clearance was obtained from the research ethical committee/institutional review board of the surgery department of Addis Ababa University (DOS/REC/59/2024/2016). Access to the collected information was limited to the principal investigator and trained data collectors. Confidentiality was maintained throughout the project.

### Data quality control

The completeness of the questionnaire was checked daily. Orientation was provided about the objective and relevance of the study, confidentiality of information, and how to extract data from logbooks and medical records. Daily supervision of the data collection process by checking the completeness and consistency of the collected data was carried out by the principal investigator.

### Data processing and analysis

The collected data were first checked for completeness and consistency before being entered into a computer. Data were coded and entered into EpiData version 4.6.1 and exported to SPSS version 29 for analysis. The chi-square test and logistic regression were applied to evaluate the association of histological patterns with their covariates, and a *P*-value <0.05 was declared statistically significant. Mean and standard deviation (SD) were used to summarize continuous variables. Categorical variables were expressed in percentage and frequency. Descriptive analysis was used for displaying, describing, and summarizing findings using texts, tables, and figures.

## Results

**Figure 1:**
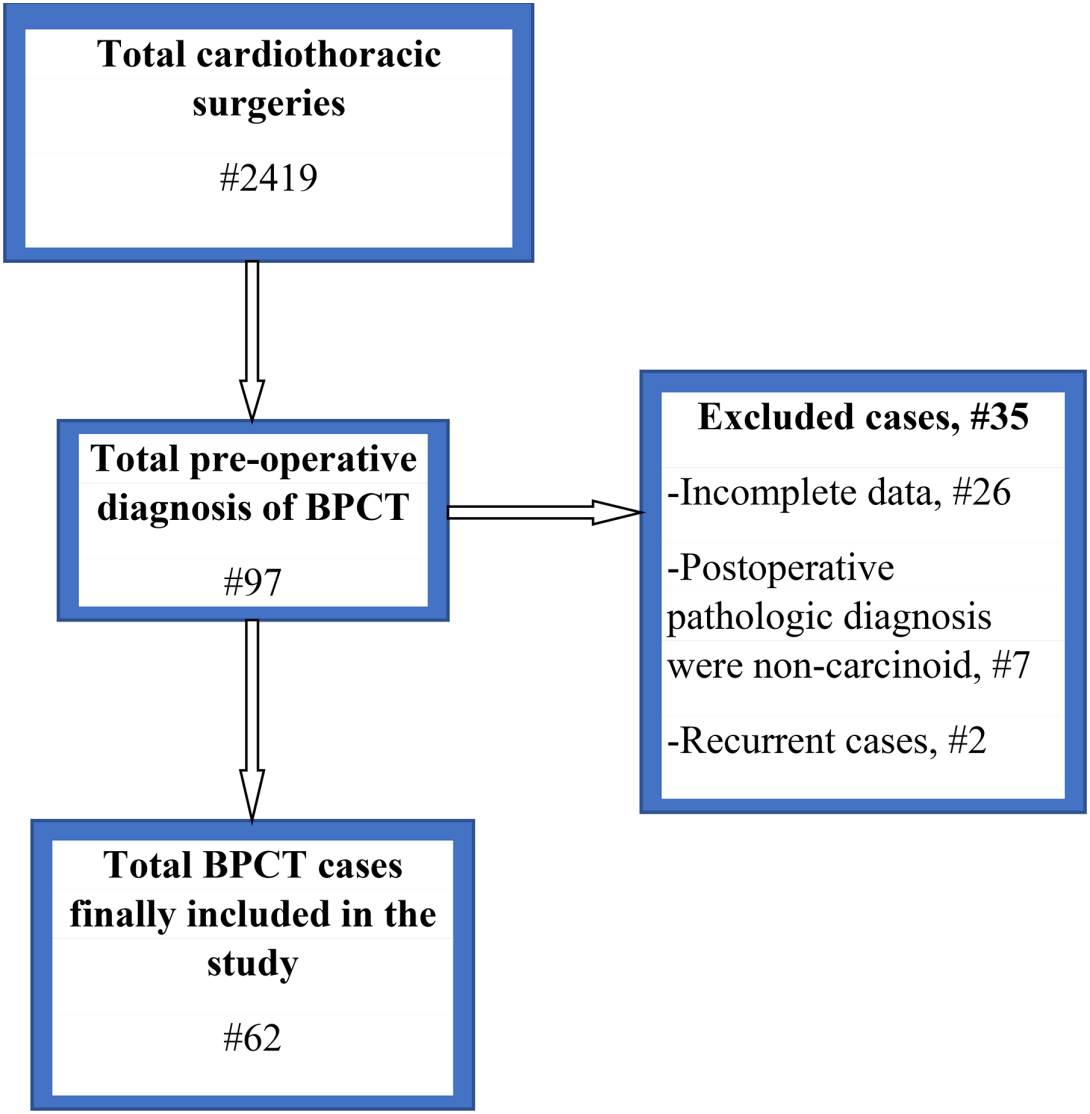
Flow chart for the characterization of study respondents. BPCT, bronchopulmonary carcinoid tumor.

From a total of 2,719 cases that were operated on for cardiothoracic surgery, 97 (3.57%) patients were operated on for a preoperative diagnosis of bronchial carcinoid at selected hospitals (74 at TASH, 17 at MH, and 6 at SPSH). Among those who were operated on at TASH, only 51 were included in the study; 5 patients had pathologic diagnoses other than carcinoid, 2 were operations for a recurrence, and 16 had incomplete data for various reasons. From MH, eight cases were included and two had other diagnoses and seven had incomplete data. Three cases were included from SPSH, and three were excluded because they had incomplete data, as displayed in Figure 1.

### Baseline characteristics of respondents

**Figure 2:**
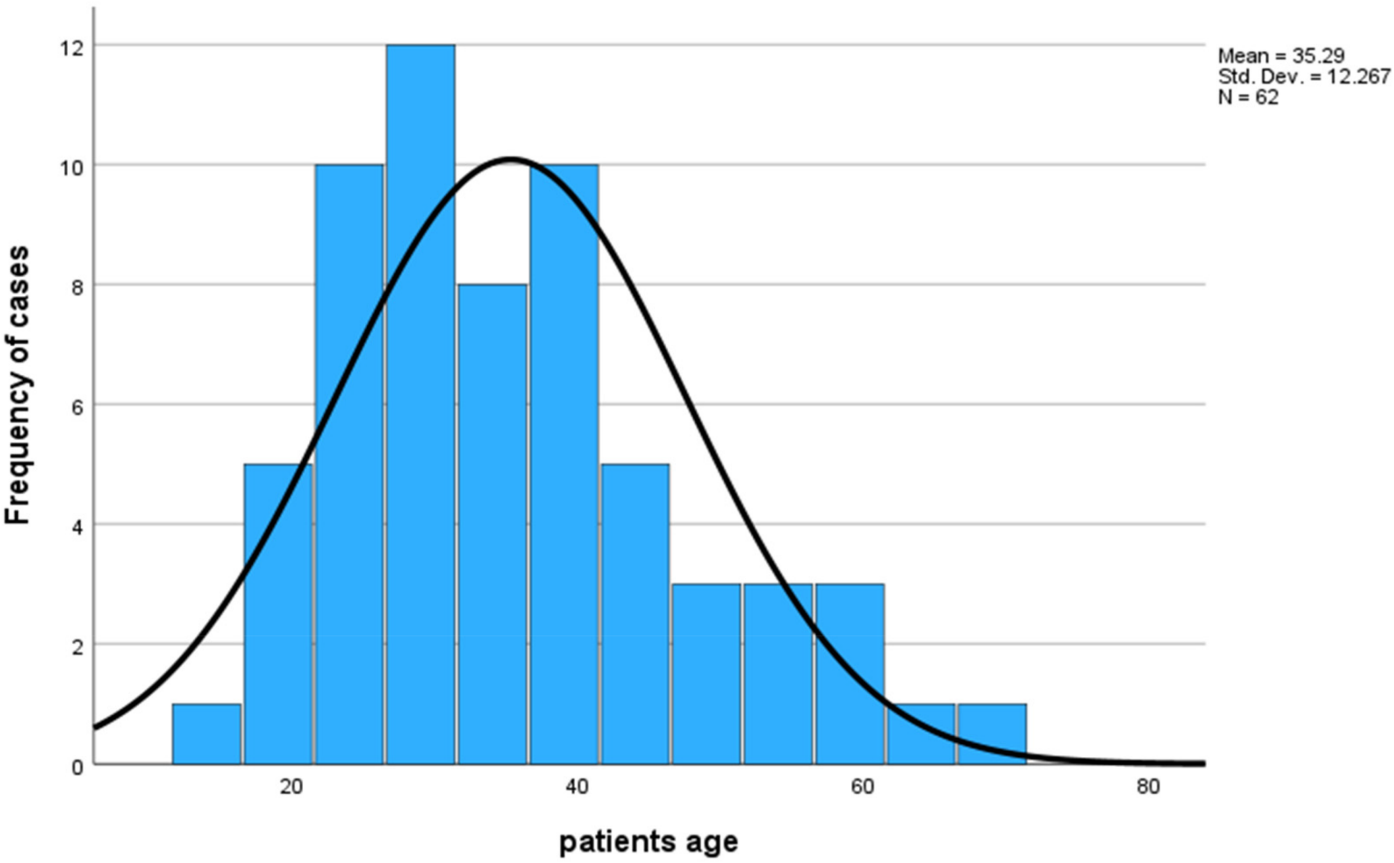
Distribution of BPCTs by age of the patients.

A total of 62 patients with BPCTs were included in the study, with a slight female predominance (56.5%) [male-to-female ratio of 27:35 (1:1.3)]. The mean age was 35.29 ± 12.26 years, ranging from 14 to 67 years (Figure 2).

### Presentation pattern

The majority of patients with BPCTs had no history of smoking [56 (90.3%)], and almost all patients were symptomatic (98.4%) during presentation. The most commonly observed symptoms were non-resolving pneumonia 47 (77%) and hemoptysis 41 (67.2%). Patients with cough accounted for 62.3%, chest pain accounted for 32.8%, and dyspnea was present in 26.2% of patients. There was no patient with carcinoid syndrome during the presentation. The mean duration of symptoms was 29.7 ± 26.13 months, ranging from 3 to 156 months (Table 1). Preoperative bronchoscopy was performed in 45 (72.6%) patients. However, bronchoscopy-guided biopsy diagnosis was carried out before surgery only for two patients (4.4%), mainly because of concerns about uncontrollable hemorrhage from the tumor manipulation. A chest CT scan was performed for all patients. The most frequently observed CT scan findings were an endobronchial mass with atelectasis of the underlying lung [21 (33.9%)] and bronchiectasis of associated lobe/s [21 (33.9%)]. Patients with a whole ipsilateral destroyed lung were 10 (16.1%) (Table 2).

**Table 1:** Clinical characteristics of the patients.

Variables		Categories	Frequency (%)
Sex		Male	25 (43.5)
		Female	37 (56.5)
History of smoking		Yes	6 (9.7)
		No	56 (90.3)
Asymptomatic		Yes	1 (1.6)
		No	61 (98.4)
Symptoms	Hemoptysis	Yes	41 (67.2)
		No	20 (32.8)
	Non-resolving pneumonia	Yes	47 (77)
		No	14 (23)
	Cough	Yes	38 (62.3)
		No	23 (37.7)
	Chest pain	Yes	20 (32.8)
		No	41 (67.2)
	Dyspnea	Yes	16 (26.2)
		No	45 (73.8)
	Carcinoid syndrome	Yes	0 (0)
		No	62 (100)
Treated for another presumptive diagnosis?		Yes	30 (48.4)
		No	32 (51.6)

Tumors were more frequently observed on the right side [34 (54.8%)] than on the left side [28 (45.2%)]. Tumors were considered “central” if they were visible in bronchoscopy or if the epicenter of the mass was within the main or lobar bronchus based on a CT scan and “peripheral” if not visible in bronchoscopy or if the mass was located distal to the lobar bronchus on a CT scan. Accordingly, in our study, 55 tumors (88.7%) were considered central and 7 (18.2%) peripheral. A destroyed lung is diagnosed when there is a total and irreversible parenchymal destruction of one lung; it is used for disease of an entire lung, not for damage to one or two lobes.

Among the central tumors, the most common site of occurrence was the left main bronchus in 14 (22.6%), followed by bronchus intermedium observed in 12 patients (19.4%). The right main bronchus tumor was observed in 11 patients (40.3%). Among the peripherally located carcinoid tumors, the left lower lobe was the most common site in 4 patients (6.5%). Based on the intra-operative descriptions, only two cases (3.2%) had extra-bronchial invasion. One of the two cases was a centrally located tumor from the right main bronchus with typical histology that extended to the mediastinal pleura. The other case was a peripherally located atypical carcinoid tumor with invasion to the diaphragm from the left lower lobe.

The tumor staging was performed based on the American Joint Committee 8th edition for Cancer Staging (39). Accordingly, most of the cases [30 (48.4%)] were categorized as T2, and T1 was observed in 6 (9.7%) cases. Only one case had T4; the one with diaphragmatic invasion. Thirty-six (58.1%) patients did not have lymph node involvement by the tumor, whereas 13 patients (21%) had confirmed lymph node involvement (either N1 or N2). For a significant proportion of cases (21%), lymph node status was not determined either because lymph node dissection was not performed and marked appropriately or it was not assessed during pathologic specimen examination. Group staging was not performed because several patients’ lymph node status was not assessed (Table 2).

**Table 2:** Tumor characteristics and preoperative workup.

Is preoperative bronchoscopy performed?		Yes	45 (72.6)
		No	17 (27.4)
Bronchoscopic biopsy was performed?		Yes	2 (4.4)
		No	43 (95.6)
Location of tumor	Central 55 (88.7)	Right main bronchus	11 (17.7)
		Left main bronchus	14 (22.6)
		Right upper lobe bronchus	5 (8.1)
		Bronchus intermedius	12 (19.4)
		Right lower lobe bronchus	1 (1.6)
		Right middle lobe	3 (4.8)
		Left upper lobe bronchus	4 (6.5)
		Left lower lobe bronchus	5 (8.1)
	Peripheral 7 (11.3)	Right upper lobe	1 (1.6)
		Right middle lobe	1 (1.6)
		Right lower lobe	0 (0.0)
		Left upper lobe	1 (1.6)
		Left lower lobe	4 (6.5)
The finding of CT scan		Isolated mass	4 (6.5)
		Atelectatic lung	21 (33.9)
		Bronchiectatic lobe/s	21 (33.9)
		Destroyed lung	10 (16.1)
		Destroyed lung with empyema	6 (9.7)
Tumor laterality		Right side	34 (54.8)
		Left side	28 (45.2)
Histologic types		TC	53 (85.5)
		AC	9 (14.5)
Extra-bronchial invasion		Yes	2 (3.2)
		No	60 (96.8)
Tumor size		T1	6 (9.7)
		T2	30 (48.4)
		T3	25 (40.3)
		T4	1 (1.6)
Lymph node involvement		NO	36 (58.1)
		N1	12 (19.4)
		N2	1 (1.6)
		NX	13 (21.0)

AC, atypical carcinoid; CT, computed tomography; TC, typical carcinoid.

Nearly half of the patients 30 (48.4%) were treated for a presumptive clinical diagnosis of pulmonary tuberculosis (PTB) before a diagnosis of carcinoid tumor was made. Some of these patients were even treated for pulmonary tuberculosis repeatedly (Table 3).

**Table 3:** Treatment for the clinical diagnosis of pulmonary tuberculosis.

PTB treatment history		Frequency	%
Not treated		32	51.6
Treated	Once	23	37.1
	Twice	7	11.3
Total		62	100

PTB, pulmonary tuberculosis.

### Surgical treatment strategies for bronchopulmonary carcinoid tumors

The majority of patients with BPCTs underwent surgery by open posterolateral thoracotomy [61 (98.4%)], and pneumonectomy was the most common (38.7%) type of resection performed. In this series, no sleeve lobectomy or sleeve pneumonectomy was performed. In three cases (4.8%), the resection margin was involved; in two of these cases, the bronchoplastic procedure was performed (Table 4).

**Table 4:** Surgical treatment strategies for bronchopulmonary carcinoid tumors.

Variables	Categories	Frequency (%)
Surgical approach	VATS	1 (1.6)
	Open posterolateral thoracotomy	61 (98.4)
Type of resection performed	Wedge resection	1 (1.6)
	Bronchoplastic procedure	5 (8.1)
	Standard lobectomy	22 (35.5)
	Sleeve lobectomy	0 (0)
	Bi-lobectomy	10 (16.1)
	Pneumonectomy	24 (38.7)
	Sleeve pneumonectomy	0 (0)
Status of resection margin	Free	59 (95.2)
	Involved	3 (4.8)

VATS, video-assisted thoracoscopic surgery.

### The association of histological patterns with other covariates

**Figure 3:**
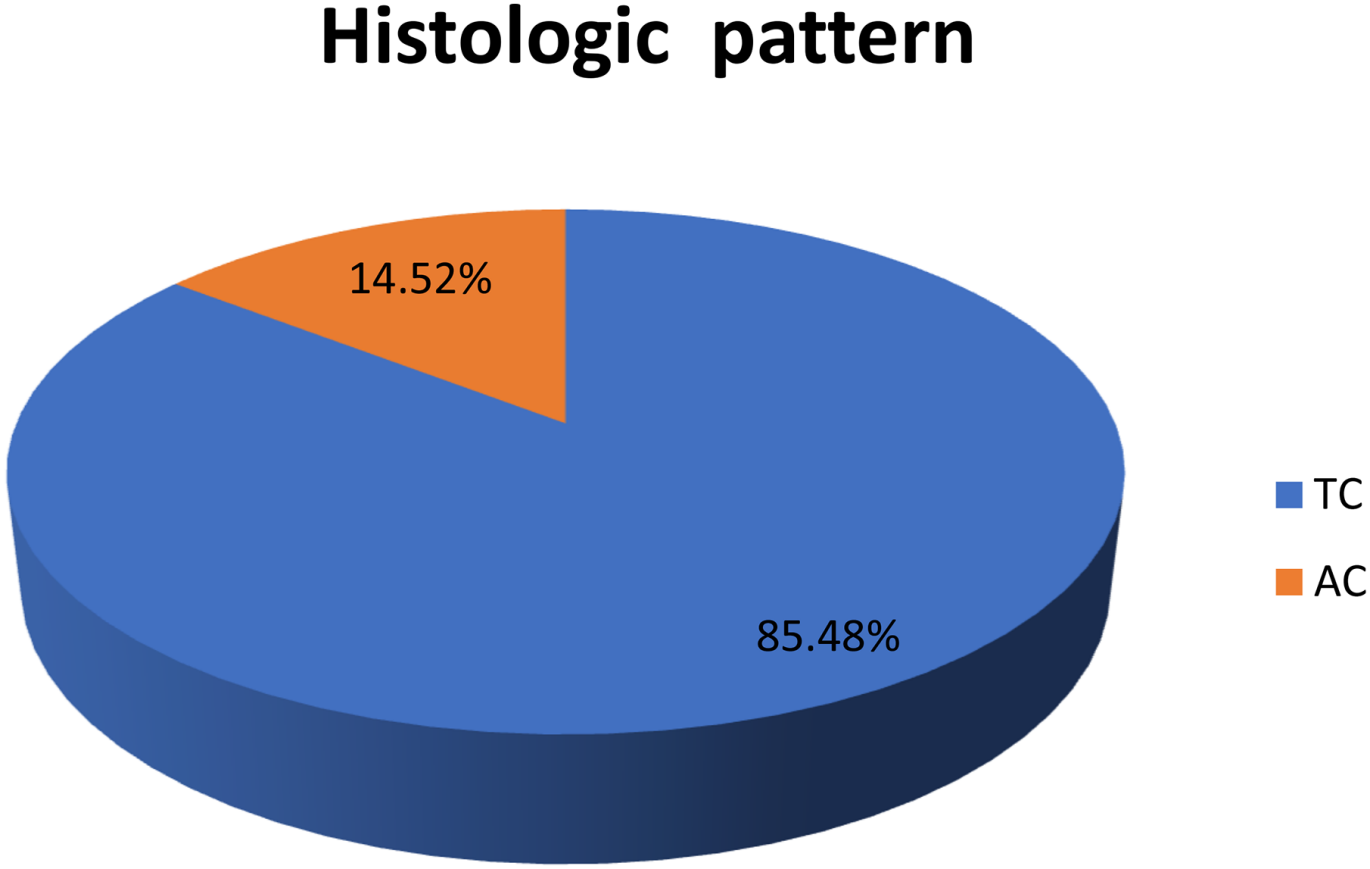
Histologic pattern of bronchopulmonary carcinoid tumors. AC, atypical carcinoid; TC, typical carcinoid.

The most common histological pattern was TC, which accounted for 85.5% of patients (Figure 3). The association of histological patterns with other covariates was evaluated using cross-tabulation and logistic regression. Accordingly, five variables (age, smoking history, duration of symptoms, location of tumors, and lymph node status) were statistically associated with histological patterns. Older patients were 1.17 times more likely to have the AC-type histological pattern [OR, 1.17 (1.07–1.29), *P*-v = 0.001]. As the duration of symptoms increased by one unit, the probability of having the AC-type histological pattern decreased 0.75 times [OR, 0.75 (0.6–0.9, *P*-v = 0.009)]. Smokers had 65 times more chance of having the AC histological pattern [OR, 65 (6–699), *P*-v = 0.001], as shown in Table 5.

**Table 5:** Association of a histological pattern of BPCTs with other covariates.

Variable	Category	Histological pattern, number (%)			
		TC	AC	OR (CI)	*P*-value
Age in years (continuous variable)		32.32 ± 10	52.78± 8.9	1.17 (1.07–1.29)	<0.001^a^
Sex	Male	22 (35.5)	5 (8.1)	1	
	Female	31 (50.0)	4 (6.5)	0.57 (0.14–2.35)	0.436
Smoking status	Yes	1 (1.6)	5 (8.1)	65 (6–699)	0.001^a^
	No	52 (83.9)	4 (6.5)	1	
Duration of symptoms in months		33.12 ± 26.8	10± 4.6	0.75 (0.6–0.9)	0.009^a^
Treatment for another presumptive diagnosis	Yes	28 (45.2)	2 (3.2)	1	
	No	25 (40.3)	7 (11.3)	3.9 (0.7–20)	0.107^a^
Location of tumor	Central	50 (80.6)	5 (8.1)	0.07 (0.01–0.4)	0.004^a^
	Peripheral	3 (4.8)	4 (6.5)	1	
Tumor laterality	Right side	30 (48.4)	4 (6.5)	0.6 (0.15–2.5)	0.501
	Left side	23 (37.1)	5 (8.1)	1	
Tumor size	T1 and T2	33 (53.2)	3 (4.8)	1	
	T3 and T4	20 (32.3)	6 (9.7)	3.3 (0.74–14.6)	0.117^a^
Lymph node status	N0	35 (71.4)	1 (2.0)	1	
	N1 and N2	7 (14.3)	6(12.2)	30 (3.1–289)	0.003^a^

AC, atypical carcinoid; OR, odds ratio; TC, typical carcinoid.

^a^ Statistically significant.

## Discussion

BPCTs are relatively rare cancers originating from neuroendocrine “Kulchitsky” cells of the bronchial tree mucosa and submucosal glands. According to the Surveillance, Epidemiology, and End Results (SEER) database, BPCTs accounted for approximately 1.2% of the 463,338 primary lung malignancies, with a rapid increment over the last three decades in the USA. A population-based retrospective cohort analysis conducted in adult patients with a NET diagnosis from 1994 to 2009 in Ontario, Canada, has also concluded there has been a marked increment in the incidence of NETs over 15 years (3, 40). The current institution-based study found that operations for BPCTs accounted for 3.57% of the total cardiothoracic surgeries performed during the study period. However, Yang et al. found that carcinoid tumors accounted for only 1% of the operations carried out for lung cancers (13). The higher proportion of surgeries conducted for BPCTs from total cardiothoracic surgeries has been attributed to the presentation of malignancies like lung cancer, commonly at stage IV according to a previous study in the same institution (41).

In our study, we found a higher proportion of female patients (56.5%) who were operated on for BPCTs, which is in line with other studies that also reported a significant female predominance (5, 8, 13). The mean age of the respondents in our study was 35.29 ± 12.26 years, ranging from 14 to 67 years, which is also in harmony with other studies.

In the current study, the majority of patients were non-smokers (90.3%) and this finding is supported by other studies (21, 22, 42). However, studies conducted in Denmark revealed a higher proportion of smokers among patients with BPCTs (5, 9). This disparity could be due to variations in population and smoking habits.

Almost all of our patients were clinically symptomatic during presentation (98.4%), which is also supported by other studies. The most frequently observed symptoms were non-resolving pneumonia and hemoptysis, which were also supported by the findings of other studies (12, 13, 21, 22, 27, 42). In addition to these symptoms, we also found dyspnea (26.2%) in our study, which was a rare presentation in other literature. This could be explained by the fact that a significant percentage of patients (25.8%) with destroyed lung were found in the current study (12, 15, 27).

The mean duration of symptoms before patients were surgically treated was 29.7 months, with a range of 3–156 months in our study. This finding is similar to a study that was carried out in Sweden by Blondal et al. from 1971 to 1976, where they found patients had symptoms that were delayed an average of 7 months, and doctor delay before treatment was on average 1.1 years (43).

In contrast to most studies, we found that almost half of the patients (48.4%) were treated for pulmonary tuberculosis empirically. This is mainly because of the lack of appropriate imaging facilities in the rural areas where most patients are referred. A lack of awareness about the non-infectious causes of respiratory symptoms among primary physicians also may have contributed.

In our case, the preoperative bronchoscopic examination was carried out for 72.6% of patients, from which bronchoscopy-guided biopsy was performed only for two patients. This finding is very low compared with other studies. In our setting, the main reason for deferring bronchoscopy-guided biopsy is concerns about uncontrollable hemorrhage, but other studies have reported a low chance of major hemorrhage following biopsy, concluding it as a safe procedure (12, 15, 27).

The most frequent radiologic findings were atelectasis (33.9%) and bronchiectasis of lobe(s) (33.9%), in which these patterns are in harmony with other studies (21, 42). In addition to these patterns, CT scans also identified destroyed lung with or without empyema in 25.8% of cases in the current study, which was not reported in other studies. These could be explained by a delayed presentation and empirical treatment for PTB, which further delays the chance of early surgery in a significant proportion of our patients.

Similar to other studies, the incidence of BPCTs was more common on the right side. In addition, we found the central location was dominant (88.7%), which is similar to studies carried out in other areas (3, 8, 12, 22, 44).

The most common histological finding in the current study was TC, which accounted for 85.5% of patients. This finding is in line with other studies (3, 22, 27, 45).

The current study found that T2 and T3 accounted for 48.4% and 40.3% patients, respectively. This finding contradicts most studies, which identified T1 as the most common presentation (1, 5, 13, 22, 45). The reason for the higher T stage in our study might be related to the late presentation of our patients after developing symptoms. In most of our cases, lymph nodes were negative for tumors in 58.1% of post-operative pathologic assessments, which is also supported by other studies. In addition, our study finding is unique in that we found NX (lymph nodes that cannot be assessed) in 21% of cases. This is probably because lymph node dissection is not performed routinely for carcinoid tumors or the lymph node is not assessed thoroughly on pathologic tissue examination.

For almost all patients, the surgical approach was open posterolateral thoracotomy in our study. This finding has a significant disparity with studies conducted in other countries. The reason we had a low number of VATS approaches is the lack of expertise and late presentation of patients with significant inflammatory adhesions, which makes the VATS approach difficult. The most common type of resection performed for BPCTs in this study was pneumonectomy, which contradicts other studies (8, 22, 27, 44, 45). The reasons we had a higher rate of pneumonectomy include a higher proportion of patients who presented with destroyed lung (25.8%) and a lack of experience in performing parenchymal sparing procedures. The fact that approximately half of the patients were treated for pulmonary tuberculosis empirically led to post-obstructive pneumonia and lung parenchymal destruction. This figure is significantly higher than those reported by European studies, in which pneumonectomy accounted for less or close to 10% in most of the studies (8, 9, 12, 27, 42, 44). As in previous studies, a negative surgical margin was achieved in most of the operated cases in this study.

Finally, five variables (age, smoking history, duration of symptoms, location of tumors, and lymph node status) were statistically associated with histologic patterns of the disease. In the current study, patients who had a history of smoking had a 65 times greater chance of developing an AC histological pattern than non-smokers. This study finding is also supported by the findings from previous studies performed in other countries (4, 13, 15, 21, 44).

The current study revealed that being older age had a 1.17 times closer association with an AC histological pattern. This finding is also similar to the study conducted in Turkey by Aydin et al., which reported a significant association between older age and an atypical histological pattern (21). Other studies also reported a higher mean age in the atypical carcinoid group than in the typical carcinoid pattern (9, 45).

Our study revealed that patients with a shorter symptom duration were more likely to have atypical histological patterns. This can be explained by the fact that atypical histology has a higher mitotic rate and rapid growth than that of a typical carcinoid (4).

We also found that carcinoid tumors located centrally were more likely to be associated with typical histological patterns. This finding is also in line with other studies (13, 21, 22, 27, 44).

Atypical carcinoids were also associated with lymph node involvement, as supported by studies performed in different settings (5, 9, 12, 27, 42, 45). The limitation of our study in lymph node staging was the fact that a significant percentage of patients had an undetermined lymph node status.

### Strengths and limitations of the study

The study describes the demographics, clinical presentations, anatomic distributions, histological types, and surgical strategies used for BPCTs in Ethiopia. It also determines the proportion of patients with carcinoid tumors who were treated for another presumptive diagnosis and describes factors influencing the histological distributions of these tumors. This study is multi-institutional, which increases the chances of including a diverse population; therefore, it has better representativeness of the general population.

This is a retrospective study extracted from a chart review, which negatively affected data quality and resulted in the exclusion of some cases because of incomplete charts. Our study also included a relatively small sample size, which affects its generalizability. The inconsistency in appropriate lymph node dissection with pulmonary resection for BPCTs has resulted in difficulty in group staging and knowing the true extent of the disease. The post-operative outcome was not included in this study, which needs to be studied with a larger sample size from different centers in the country.

## Conclusion

There was a slight female predominance (56.5%), with the mean age of patients being 35.29 years. The majority of the patients had no history of smoking (90.3%) and most of them were symptomatic at presentation (98.4%). The majority of the patients underwent surgery by open posterolateral thoracotomy (98.4%) and pneumonectomy was the most frequent (38.7%) resection performed. The most common histological pattern was TC, which accounted for 85.5% of patients. Age, smoking history, duration of symptoms, location of tumors, and lymph node status were statistically associated with histologic patterns.

We found nearly half of the patients were treated for PTB before surgical treatment for carcinoid tumors. This empirical treatment causes delays in patient presentation, leads to unnecessary costs, and exposes patients to adverse reactions to anti-TB drugs. This is probably a factor responsible for the significantly higher proportion of pneumonectomies, which cause more morbidity than resections, which are less common. Therefore, all physicians should think of alternate diagnoses and investigate patients appropriately if a patient is negative for routine TB workup or has an inadequate response to appropriate antibiotics.

The most common surgical approach was open posterolateral thoracotomy, which is invasive for early tumors without extensive intrathoracic adhesion. Thus, we recommend that institutions provide facilities to undergo VATS and prepare surgeons to perform this relatively less invasive procedure for appropriate cases. During surgical treatment for carcinoid tumors, the lymph nodes should be dissected routinely and stations marked for the pathologists to complete the staging. Thus, the pathologist should also assess all lymph nodes to stage the disease of the patient well. In conclusion, we recommend that researchers should study this topic further.

## Data Availability

The original contributions presented in the study are included in the article/Supplementary Material, further inquiries can be directed to the corresponding author.
